# Metal Organic Frame-Upconverting Nanoparticle Assemblies for the FRET Based Sensor Detection of Bisphenol A in High-Salt Foods

**DOI:** 10.3389/fbioe.2020.626269

**Published:** 2020-12-22

**Authors:** Zhou Xu, Lin-wei Zhang, Ling-li Long, Shao-hua Zhu, Mao-long Chen, Li Ding, Yun-hui Cheng

**Affiliations:** ^1^College of Chemistry and Food Engineering, Changsha University of Science & Technology, Changsha, China; ^2^Changsha Customs Technology Center, Changsha Customs District P.R. China, Changsha, China

**Keywords:** fluorescence resonant energy transfer, metal organic frame-upconversion nanoparticle assembly, high-salt foods, bisphenol A, aptamer

## Abstract

To resolve the occurrence of unfulfillable detection in high-salts foods, we used fluorescence resonant energy transfer (FRET) sensors based on nanoparticle upconversion. In this study, we developed a novel FRET sensor for the detection of bisphenol A (BPA) in high-salt foods. We based this approach on the assembly of aptamer modified upconversion nanoparticles (DNA1-UCNPs) and complementary DNA modified metal organic frames (DNA2-MOFs), which possessed corresponding wavelength absorption. Targeting BPA signal transduction was performed using the BPA aptamer, via competitive recognition between the BPA analyte and complementary DNA sequences in a high-salt solution. Sensor adaption in high-salt samples was attributed to functional hydrophilic groups, modified in the MOFs, and the enhanced colloidal stability of these MOFs. The MOF-UCNP assembly displayed considerable analytical performance in terms of BPA detection, with a linear range of 0.1–100 nM, and a limit of detection (LOD) of 0.02 nM, in a 340 mM NaCl food sample (the energy drink, Gatorade). Thus, this method provides a solid basis for small molecules detection in high-salt foods.

## Introduction

In recent years, upconversion nanoparticle (UCNP) based fluorescence resonant energy transfer (FRET) sensors (FBUFS) have gained traction as tools for various applications, including bioassays and sensing (Li et al., 2018; Yan et al., 2018; Soni et al., 2019; Wang et al., 2019). For applications in the detection field, these FRET sensors are used to detect small organic molecules (Lo et al., 2020), peptides (Duvall and Childers, 2020), proteins (Liu et al., 2014), ions (Wang et al., 2019), or cells and bacteria (Hwang et al., 2019). However, their application to high-salt foods has been largely limited due to their poor colloidal stability in these foods. Thus, to make FRET-based upconversion fluorescence sensors (FBUFS) more appropriate for high-salt foods, there is an urgent need to improve FBUFS stability.

A traditional FBUFS is formed between UCNPs and gold nanoparticles (AuNPs), with the UCNPs acting as the donor, and the AuNPs as the acceptor (Baek et al., 2018). UCNPs possess excellent colloidal stabilities under salt high concentrations, due to the presence of hydrophilic groups and the steric repulsion of hydrophilic polymers (Yao et al., 2014; Ulusoy et al., 2016). For AuNPs, aggregates are rapidly formed in high salt solutions thanks to decreased electrostatic repulsion, induced by the high salt environment (Sun et al., 2020; Zhang et al., 2020). Therefore, novel acceptors with ultra-high colloidal stabilities, especially in high salt solutions, will improve the performance of FBUFS in high salt food samples.

Metal-organic frameworks (MOFs) are novel functional porous materials consisting of metal nodes and organic linkers, and providing MOFs with a mass of functional groups (He et al., 2019; Chen et al., 2021; Xu et al., 2021). These functional hydrophilic groups can be further modified to enhance MOF colloidal stability (Bellido et al., 2014; Kim et al., 2019). In high salt solutions, hydration layers between functional hydrophilic groups weaken electrostatic repulsion induced by high salt, this limiting salt-induced aggregation and facilitating the high colloidal stability of MOFs in these solutions. In addition, because of the huge diversity of organic linkers, MOFs possess tunable UV-Vis spectra capabilities resulting from ligand functionalization, and different combinations of metal nodes and organic linkers (Espin et al., 2018; Wang et al., 2020). Accordingly, these tunable UV-Vis spectra can be overlapped with emission spectra from UCNPs, inducing fluorescence resonant energy transfers between UCNPs and MOFs. Recently, several MOFs, such as MIL-88A (Tan et al., 2016) and ZIF-8 (Hao et al., 2019) were used as quenchers for DNA detection. When compared with traditional quenchers i.e., AuNPs, MOFs exhibit considerable fluorescence quenching abilities. Taken together, MOFs is a potential acceptor under water with high salt content.

**FIGURE 1:**
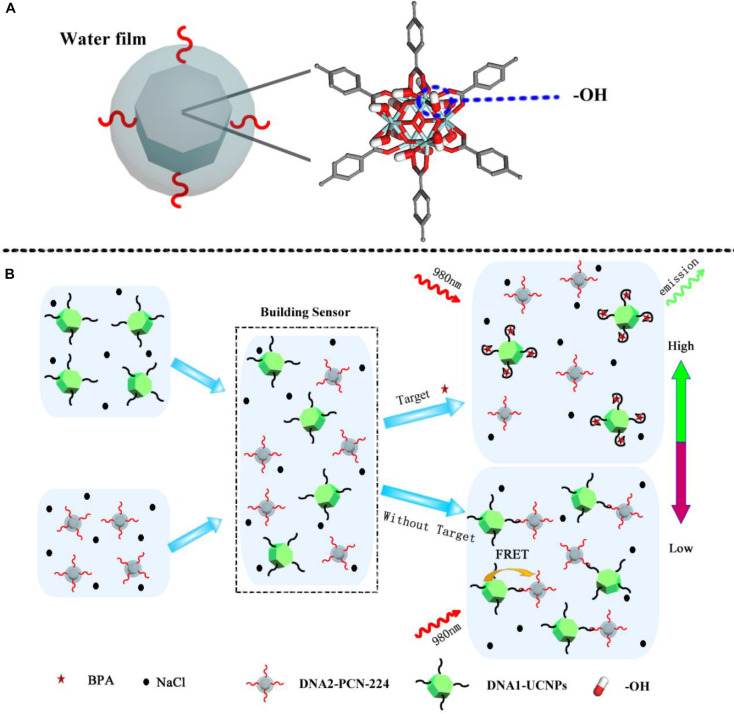
**(A)** Schematic illustration of molecular structure of the MOFs. **(B)** MOFs-UCNP platform assembly for the detection of BPA in a high salt solution.

In this study, we established a new optical nanosensor using MOF assembly, for the high-salt detection of small molecules. As a model analytical small molecule, we decided to use bisphenol A (BPA), in view of its significant impact on human health. The MOF, PCN-224 was selected as the acceptor in the nanosensor. MOFs possess colloidal stabilities thanks to their free hydrophilic -OH groups (Figure 1A). As shown (Figure 1B), when a BPA aptamer-modified UCNP (DNA1-UCNP) was mixed in solution with complementary DNA sequences of the complementary DNA-coated MOF (DNA2-MOF), a recognition process, driven by the coupling reaction, promoted the assembly of the MOF-UCNP complex. Similarly, fluorescence intensity values were decreased due to FRET between adjacent plasmonic MOFs and UCNPs. When the analyte BPA was added to this nanosensor, the aptamer of BPA competitively recognized the analyte BPA and complementary DNA sequences in solution, which maintained the dispersed state of DNA1-UCNP and DNA2-MOF. Our research suggests our nanosensor could be used as a facile, highly sensitive and selective analytical tool to detect BPA in high-salt foods.

## Materials and Methods

### Chemicals and Materials

The following chemicals were purchased from Beijing Bailingwei Technology Co. Ltd, China; yttrium (III) chloride hexahydrate (YCl_3_⋅6H_2_O, 99.99%), ytterbium (III) chloride hexahydrate (YbCl_3_⋅6H_2_O, 99.9%), erbium (III) chloride hexahydrate (ErCl_3_⋅6H_2_O, 99.9%), oleic acid (OA > 85%), 1-octadecene (1-ODE, 90%), N-(3-Dimethylaminopropyl)-N’-ethylcarbodiimide hydrochloride (EDC, 99%), N-hydroxysuccinimide (NHS, 98%), zirconium dichloride oxide octahydrate (ZrOCl_2_⋅8H_2_O, 98%), poly (acrylic acid) [PAA, Molecular weight (MW) = 5,000], bisphenol A (BPA, 96%), 4-cumylphenol (4-CP, 99%), bisphenol B (BPB, 99%), 4-octylphenol (4-OP, 99%) and bisphenol A glycidyl ether (BADGE, 98%).

The following chemicals were purchased from Sinopharm Chemical Reagent Co., Ltd. (Shanghai, China); sodium hydroxide (NaOH), ammonium fluoride (NH_4_F), methanol, benzoic acid (BA), cyclohexane, N, N-dimethylformamide (DMF), and Sodium chloride (NaCl).

The following chemical were purchased from Beijing Huawei Ruike Chemical Co., Ltd., China; Meso-tetra (4-carboxyphenyl) porphine. The following chemical were purchased from Chengdu Cologne Chemical Co. Ltd, China; Trichloromethane.

DNA oligonucleotides were purchased from Sangon Biotech (Shanghai, China) Co. Ltd., at a concentration of 100 nM. The oligonucleotide sequences were:

DNA1 (aptamer of BPA):

5′-NH_2_-TTTTTTTTTTCCGGTGGGTGGTCAGGTGGGA TAGCGTTCCGCGTATGGCCCAGCGCATCACGGGTTCGC ACCA-3′.

DNA2 (complementary DNA sequences):

5′-NH_2_-TTTTTTTTTTCCCACCTGACCACCCACCGG-3′.

### Equipment and Apparatus

Fluorescence experiments were carried out by F96PRO Fluorespectrophotometer (Shanghai Lingguang Technology Co., Ltd.) with a gain of 8, spectra were recorded at the range of 500–700 nm under 980 nm excitation. UV-vis absorption spectrum were identified with UV-1800 UV-visible spectrophotometer (Shimadzu, Japan) and the sample was placed in 1 cm path length quartz cuvettes. The size of the formed nanomaterial by a dynamic laser light scattering instrument (DLS) using a Malvern Zetasizer nano ZS90 (Malvern Instruments), the samples were dispersed in water and measured for three times under the room temperature. Transmission electron micrographs were taken on a JEOLJEM-2100 transmission electron microscope. Fourier-transform infrared spectroscopy (FTIR) spectra analyses was conducted on a Spectrum GX (Perkin-Elmer, United States).

### Preparation of UCNPs

Mono-dispersed UCNPs were prepared by an established solvothermal method, according to previous studies, but with slight modifications (Tian et al., 2018; Jin et al., 2019). An OA (6 mL), 1-ODE (15 mL) and 1 mmoL rare earth Chloride complexes (comprising 78% Y^3+^, 20% Yb^3+^, and 2% Er^3+^) were added to a 100 mL three-necked flask. The mixture was heated to 160°C under nitrogen, until a clear solution was formed. A 30 mL methanol solution containing NaOH (3.25 mmoL) and NH_4_F (3.5 mmoL) was then added drop-wise to this solution, and stirred for an additional 30 min after the reaction cooled to 80°C. To remove the methanol, the solution was stirred at 100°C for 30 min. After this, the solution was heated to 300°C and maintained at a constant temperature, under nitrogen. The solution was then cooled to 25°C after 1 h stirring. An ethanol and cyclohexane mixture in a 1:1 ratio was then added to the solution and centrifuged at 4,500 rpm for 10 min at 25°C. The UCNPs, coated by OA, were washed twice in ethanol, and 2–3 times in water. Then, the UCNPs coated with OA were dispersed in chloroform. After this, a 10 mL chloroform solution containing UCNPs (50 mg) was added to a 50 mL flask. A 20 mL ethanol solution containing PAA (300 mg) was then slowly added to the flask. After this, the solution was stirred for 24 h at 25°C. UCNPs coated in PAA were then obtained by centrifugation, and dispersed in water.

### Preparation of MOFs

The MOF, PCN-224 was prepared according to a previous method, but with slight modifications (Gong et al., 2019). Approximately 0.1 g H_2_TCPP, 0.3 g ZrOCl_2_⋅8H_2_O, 2.2 g BA and 60 mL DMF were fully dissolved in a 150 mL Erlenmeyer flask. The solution was heated to 90°C, and stirred for 5 h. After cooling to room temperature, PCN-224 was collected by centrifugation, and washed three times in DMF. The “as-prepared PCN-224” was then dispersed in water.

### Preparation of DNA1-UCNPs

According to a previous study (Raj et al., 2015), 2.53 mg UCNPs were dispersed in 0.79 mL ultra-pure water, and reacted with 100 μL EDC (15 mg/mL). After 15 min stirring, 100 μL NHS (20 mg/mL) was added, and allowed react for 2 h, with continuous stirring. The solution was centrifuged and the UCNPs were dispersed into 0.99 mL ultra-pure water. After this, 10 μL DNA1 (100 nM) was added into the UCNP solution and reacted for 8 h, with continuous stirring. The DNA1-UCNPs were collected by centrifugation, and dispersed in ultra-pure water (1 mL).

### Preparation of DNA2-MOFs

DNA2-modificated MOFs were prepared according to a previous study, but with slight modifications (Raj et al., 2015). Briefly, 0.63 mg PCN-224 was dispersed in 0.79 mL ultra-pure water. To this, 100 μL EDC (15 mg/mL) was added, and the solution allowed react for 15 min, with slow stirring. After this, 100 μL NHS (20 mg/mL) was added, and the solution allowed react for 2 h, with continuous stirring. Afterward, the solution was centrifuged and the PCN-224 dispersed into 0.99 mL ultra-pure water. To this, 10 μL DNA2 (100 nM) was added and reacted for 8 h, with continuous stirring. The “as prepared DNA2-PCN-224” was collected by centrifugation and dispersed in ultra-pure water. To avoid electrostatic interactions between UCNPs and MOFs, the “as-prepared DNA2-PCN-224” was modified with Bovine serum albumen (BSA). Briefly, a water solution (10 mL) containing DNA2-PCN-224 (5 mg) was added to 10 mL water containing BSA (10 mg/mL). After 30 min stirring, DNA2-PCN-224@BSA was obtained by centrifugation. DNA2-MOFs were washed three times and dispersed in ultra-pure water. DNA2-PCN-224@BSA was then replaced by DNA2-PCN-224.

### Detection of BPA

For BPA detection, 500 μL 2 nM DNA1-UCNPs were added to a tube, and reacted with 10 μL BPA, in a series of concentrations ranging from 0–1,000 nM. After a 60 min reaction at room temperature, 490 μL of 32 nM DNA2-PCN-224 was added to the solution. This solution was then placed in a micro cuvette after a 60 min incubation. To investigate the effects of BPA on signal recovery, a F96PRO fluoro-spectrophotometer was used to analyze sensor signal intensity at 543 nm. All experiments were repeated three times.

## Results and Discussion

### Synthesis, Assembly, and Characterization of MOFs-UCNPs

**FIGURE 2:**
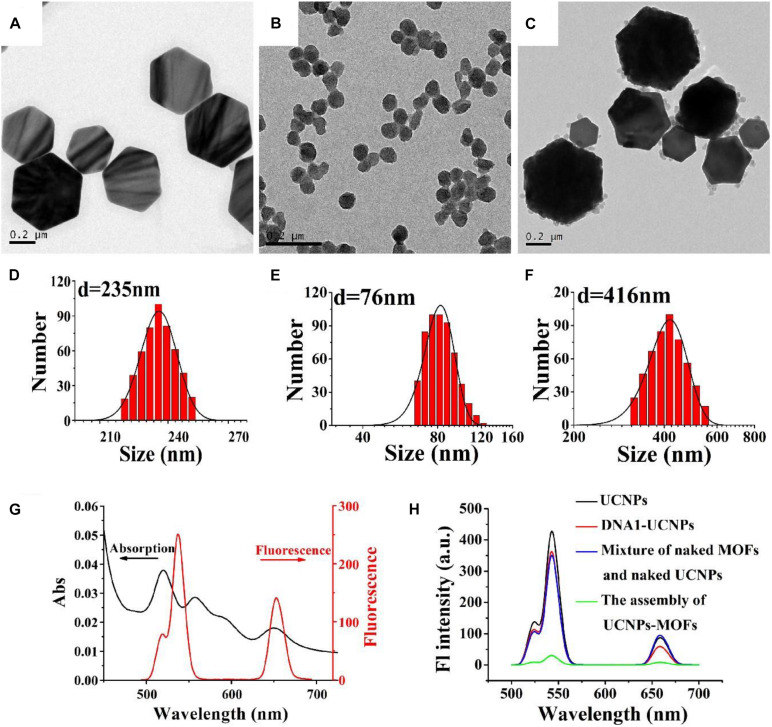
TEM images of DNA1-UCNPs, DNA2-MOFs, and MOFs-UCNP assembly **(A–C)**. Size distributions of DNA1-UCNPs DNA2-MOFs and MOFs-UCNPs assembly **(D–F)**. Absorption spectrum of MOFs and the emission spectrum of UCNPs **(G)**. The fluorescence spectra of UCNPs (black line), DNA1-UCNPs (red line), mixture of naked MOFs and naked UCNPs (blue line), MOFs-UCNPs assembly (green line) **(H)**.

The size distribution, shape and assembly structures of DNA1-UCNPs, DNA2-MOFs, and MOFs-UCNPs were characterized by dynamic light scattering (DLS), and transmission electron microscopy (TEM). To perform assays, DNA1-UCNPs and DNA2-MOFs were formed between nanoparticles and DNA, by the addition of EDC and NHS. As shown (Figures 2A,B), TEM revealed that DNA1-UCNPs and DNA2-MOFs possessed excellent dispersal capacities. Also, the “as prepared DNA1-UCNPs and DNA2-MOFs” had average hydro-diameters of approximately 235 and 76 nm, respectively (Figures 2D,E). The average hydro-diameter of DNA1-UCNPs and DNA2-MOFs were larger than the actual diameters of that, which were similar to previous reports (Dibaba et al., 2018; Zhang et al., 2019). As shown (Figure 2C), MOFs-UCNP assembly was successfully established by the addition of DNA1-UCNPs and DNA2-MOFs. As shown (Figure 2F), following the addition of DNA2-MOFs, the sensor hydro-diameter increased to 416 nm, indicating full MOFs-UCNP assembly.

To demonstrate the feasibility of PCN-224 as an acceptor of the sensor, fluorescence and absorption spectra were gathered. Figure 2G shows the absorption spectrum of MOFs, and the emission spectra of UCNPs. We observed that the broad absorption band (500–700 nm) of MOFs largely overlapped with the emission spectra (543 nm) of UCNPs. This indicated that FRET had occurred between UCNPs and MOFs. It was important to observe the fluorescence intensity of MOFs-UCNP nanostructures before and after assembly, as they had changed considerably (Figure 2H). As the MOFs-UCNP assembly formation progressed and caused fluorescence to disappear, this was attributed to FRET between adjacent plasmonic MOFs and UCNPs.

### The Colloidal Stability of UCNPs, MOFs, and AuNPs

**FIGURE 3:**
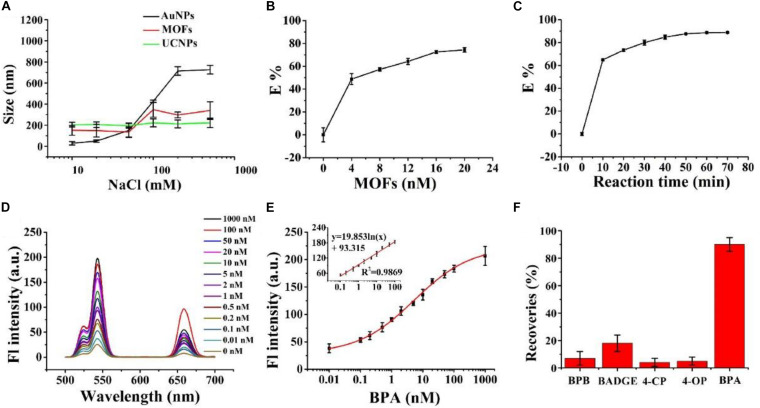
**(A)** The effects of different NaCl concentrations on the hydrodynamic diameters of AuNPs (black line), PCN-224 (red line), and UCNPs (blue line). **(B)** The quenching efficiency of UCNPs toward different MOF concentrations. **(C)** The quenching efficiency of reaction time. **(D)** Fluorescence spectra of the MOFs-UCNPs assembly with different BPA concentrations. The excitation wavelength was fixed at 980 nm. **(E)** Fluorescence intensities at 543 nm were plotted against 0.01–1,000 nM BPA concentrations. The inset shows the linear calibration plot between the recovered fluorescence intensity of the MOFs-UCNPs assembly and BPA concentrations from 0.1–100 nM. **(F)** Recoveries of spiked BPA and BPA analogs in water, following the assembly of MOFs-UCNPs. The concentrations of BPA and BPA analogs were 0.1 and 1 nM, respectively.

We used stability assays to assess high-salt food adaptability of the nanosensor. MOFs and UCNP stability was evaluated in NaCl solutions at different concentrations, and compared with AuNPs. Figure 3A shows a graph of MOFs, AuNPs, and UCNP hydrodynamic diameters vs. NaCl solutions at different concentrations (10–500 mM); MOFs displayed a greater stability, with no noticeable size increases after 60 min incubation. This was due to large free –OH groups of the Zr6 cluster. Also, UCNPs possessed considerable colloidal stability, with no obvious changes in size.

Similarly, we also tested traditional acceptors, AuNPs, for stability in high-salt solutions. We observed that AuNP sizes increased considerably in line with increasing NaCl concentrations, specifically in the 50–200 mM range. These data indicated that MOFs and UCNPs possessed considerable colloidal stability at high salt concentrations, ranging from 0–500 mM, and had improved salt-resistance when compared to AuNPs.

### Optimization of Assembly Conditions

To achieve an optimal detection performance, a series of experimental conditions were optimized. First, the influence of MOF concentrations on quenching efficiencies were investigated. This showed that quenching efficiencies increased with increasing MOF concentrations (Figure 3B). When the DNA2-MOFs concentration reached 4 nM, the quenching efficiency increased slowly, and then achieved equilibrium. Subsequently, the quenching efficiency was at 78% when the MOFs-DNA2 concentration reached 16 nM. These data suggested that a lower MOFs-DNA2 concentration could not completely quench the fluorescent intensity of UCNPs, thus inducing unnoticeable fluorescence restoration changes, therefore we selected 16 nM as the optimal MOFs-DNA2 concentration for subsequent assays. We also investigated the influence of nanosensor incubation times on the quenching efficiency (Figure 3C). The quenching efficiency increased with increasing incubation times, and reached a plateau at 50 min. Therefore, this incubation time was adopted for subsequent experiments.

### The Detection of BPA

To demonstrate the feasibility of the MOFs-UCNP assembly for BPA detection, we tested assembly responses to different BPA concentrations, in a high-salt solution (500 mM NaCl). As shown (Figures 3D,E) at 543 nm, the fluorescence intensity of the nanoprobe gradually increased with BPA concentration, increasing from 0 to 1,000 nM. A good linear relationship was observed between fluorescence intensity and BPA concentrations, over the 0.1–100 nM range. This method also showed a good correlation coefficient, *R*^2^ = 0.9869 within the linear detection range 0.1–100 nM, and an LOD of 0.02 nM. These data were in agreement with other methods, such asfluorescence assays (Lee et al., 2017), electrochemistry (Alam and Deen, 2020), enzyme linked immunosorbent assay (ELISA) (Kuang et al., 2014) and high performance liquid chromatography (HPLC) (Ousji et al., 2020).

### Real Sample Assessment

To study nanosensor feasibility and applicability, it was used to measure BPA levels in a real sample of high ionic strength. Gatorade is an energy drink containing 340 mM NaCl, and was selected as a model beverage for testing. As shown (Table 1), the limit of detection (LOD) of the nanosensor (0.02 nM) was lower than that of the National Food Safety Standard of China at 0.6 mg/kg (2.63 μM) (Gallo et al., 2017). The average recovery of the MOFs-UCNP sensor and the traditional fluorescence sensor, AuNPs-UCNP was 107 and 0%, respectively. These data suggested our novel fluorescence sensor demonstrated an increased high-salt stable when compared to traditional fluorescence sensors, and could be employed as a quantitative tool for BPA detection in high-salt food samples.

**TABLE 1:** BPA recoveries in Gatorade; comparisons between our novel fluorescence sensor and a traditional fluorescence sensor.

**Sample**	**Original (nM)**	**Added (nM)**	**Detected (nM)**			
			**Our nanosensor (MOFs-UCNP)**	**Recovery (%)**	**Traditional fluorescence sensor (AuNPs-UCNP)**	**Recovery (%)**
Gatorade	0	1	1.02 ± 0.07	102	0	0
	0	5	5.84 ± 0.25	117.3	0	0
	0	20	22.43 ± 0.51	102.7	0	0

### Specificity Applications

MOFs-UCNP assembly specificity was also investigated by comparing fluorescence signal responses for BPA (0.1 nM) with BPA analogs, including 4-OP, 4-CP, BADGE, and BPB at 1 nM. As shown (Figure 3F), the fluorescence intensity recovery of the nanosensor in the presence of BPA analogs was <30%, which was considerably lower that BPA (85–95%). These data indicated the nanosensor exhibited greater specificity and selectivity for BPA alone.

## Conclusion

We demonstrated that the MOF, PCN-224 served as an efficient quencher for the FRET assay for BPA due to its excellent colloidal stability under high NaCl conditions. The MOF exhibited absorption bands at 500–700 nm, allowing us to match its absorption spectrum with the emission spectrum of UCNPs, thereby producing a satisfying quenching efficiency of approximately 90%. The excellent colloid stability of the nanosensor was due to large free -OH groups scattered around the Zr6 cluster of the MOF. Our targeted BPA analyses indicated the LOD of the MOFs-UCNP assembly was 0.02 nM. These results suggested our MOFs-UCNP assembly had an excellent colloidal stability and good analytical performance, and was capable of detecting minute BPA levels, which fabricated to detect BPA in high-salts foods.

## Data Availability Statement

All datasets generated for this study are included in the article/Supplementary Material, further inquiries can be directed to the corresponding authors.

## Author Contributions

ZX: conceptualization and writing—review and editing. LZ: methodology and writing—original draft. LL: supervision and validation. SZ: validation. MC: project administration. YC: project administration and writing—review and editing. All authors contributed to the article and approved the submitted version.

## Conflict of Interest

The authors declare that the research was conducted in the absence of any commercial or financial relationships that could be construed as a potential conflict of interest.
