# Supplementary material for: Metal Organic Frame-Upconverting Nanoparticle Assemblies for the FRET Based Sensor Detection of Bisphenol A in High-Salt Foods
Source: Front Bioeng Biotechnol. 2020 Dec 22;8:626269. doi: 10.3389/fbioe.2020.626269 (PMC7783312; doi:10.3389/fbioe.2020.626269)
Supplement: Supplementary file 1 [file Data_Sheet_1.docx]

**Supporting information**

***Metal organic frame-upconverting nanoparticle assemblies for the FRET based sensor detection of bisphenol A in high-salt foods***

**Zhou Xu ^a*^, Lin-wei Zhang ^a^, Ling-li Long ^a^ , Shao-hua Zhu ^b*^ , Mao-long Chen ^a^ , Li Ding ^a^ ,Yun-Hui Cheng ^a*^**

^a^College of Chemistry and Food Engineering, Changsha University of Science & Technology, Changsha 410114, China

^b^Changsha Customs Technology Center, Changsha Customs District P.R. China, Changsha 410114, China

*Correspondence: Zhou Xu

Email: xz_jnu@126.com

Yunhui Cheng

Email: [chengyh6488@gmail.com](mailto:chengyh6488@gmail.com)

Tel: +86-731-85258322

Shao-hua Zhu

Email: ishaohua@foxmail.com

**Keywords:** *fluorescence resonant energy transfer (FRET), metal organic frame-upconversion nanoparticle (MOF-UCNP) assembly, high-salt foods, bisphenol A (BPA)*


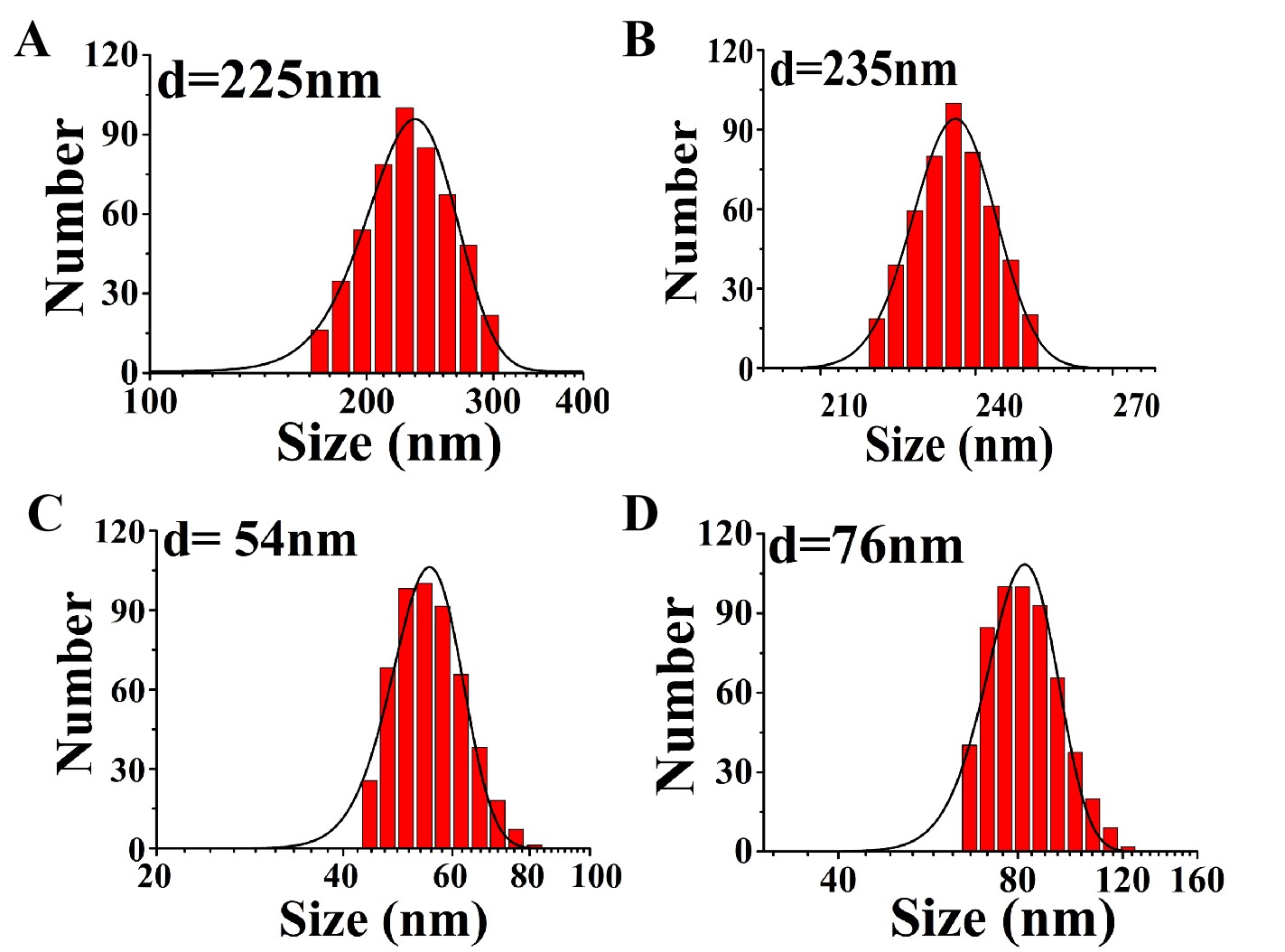


**Supplementary Figure 1.** (A-D) DLS analysis of UCNPs, DNA1-UCNPs, MOFs and DNA2-MOFs

***1. DLS analysis of UCNPs, DNA1-UCNPs, MOFs and DNA2-MOFs***

For the purpose of certifying DNA1 and DNA2 were successfully attached to the surface of UCNPs and MOFs, the DLS experiment was performed. The diameters of UCNPs, DNA1-UCNPs, MOFs, and DNA2-MOFs were 225 nm, 235 nm, 54 nm, and 76 nm, respectively. Compared with the diameters of UCNPs and MOFs, the diameters of DNA1-UCNPs and DNA2-MOFs from DLS analysis increased about 10 nm and 22 nm, respectively. The increase in the diameters indicated that the successful immobilization of DNA1 and DNA2 onto the surface of the nanoparticles, which were similar to previous reports (Shiao et al., 2014).

Shiao, Y.-S., Chiu, H.-H., Wu, P.-H.Huang, Y.-F. (2014). Aptamer-Functionalized Gold Nanoparticles As Photoresponsive Nanoplatform for Co-Drug Delivery. *ACS Appl Mater Interfaces, 6*(24), 21832-21841. [10.1021/am5026243](https://doi.org/10.1021/am5026243)
